# Targeted Temperature Management for Traumatic Asphyxia: A Case Report

**DOI:** 10.7759/cureus.55683

**Published:** 2024-03-06

**Authors:** Yoshiaki Yoshioka, Kenta Mitsusada, Go Makishi, Kazuhiro Shiga, Tatsuya Hayakawa

**Affiliations:** 1 Department of Emergency Medicine, Seirei Mikatahara General Hospital, Hamamatsu, JPN; 2 Department of Surgery, Seirei Hamamatsu General Hospital, Hamamatsu, JPN; 3 Department of Medicine, Trauma and Acute Critical Care Medical Center, Tokyo Medical and Dental University Hospital, Tokyo, JPN

**Keywords:** thoracic trauma, trauma critical care, emergency critical care, ttm, targeted temperature management, traumatic asphyxia

## Abstract

Traumatic asphyxia (TA) is a rare condition due to severe crush injury to the upper abdomen or chest region. Elevated intrathoracic pressure causes impaired venous return, which damages the small vessels. Consciousness is reportedly lost in many TA cases. In the most severe cases, hypoxic encephalopathy occurs. Since TA patients usually have other traumatic complications such as thoracic or abdominal injury, the mortality rate of this syndrome is quite variable.

Hypothermia is a risk factor for mortality in trauma patients, and targeted temperature management (TTM) is rarely performed for trauma cases. There are scattered articles reporting the usefulness of TTM in severe traumatic brain injury. To our best knowledge, there have been no reports of TTM in TA cases. We herein report a TA case with decorticate rigidity having a good neurological outcome after TTM.

## Introduction

Traumatic asphyxia (TA) is a rare syndrome resulting from strong compression on the thorax or upper abdomen. Since intrathoracic pressure is elevated, breathing and venous return are impaired, and small vessels are collapsed. Cerebral perfusion can also be impaired. The symptoms are swelling or petechiae around the face and neck, bilateral periorbital ecchymosis, hyperemia or hemorrhage of the ocular conjunctiva, and ecchymoses over the upper thorax [1]. TA patients often have other traumatic complications including thoracic or abdominal injury. Thus, the mortality rate of this syndrome is quite variable [2].

In TA patients, consciousness is reportedly lost in more than 30% of the patients [3,4]. Cerebral perfusion is also impaired leading to hypoxic encephalopathy in the most severe cases. The neurological prognosis of TA is defined by the degree of hypoxemia and brain damage. To maintain cerebral function, targeted temperature management (TTM) is performed in post-cardiac arrest patients [5]. However, it is rare to perform TTM for trauma patients because hypothermia leads to coagulopathy and there is not enough evidence. There are some papers reporting the effectiveness of TTM for brain injury including traumatic cases [6,7]. They suggested that fever was associated with adverse outcomes and TTM might be effective especially for the ischemia-reperfusion pathophysiology of traumatic brain injury (TBI).

Due to the small number of cases of TA, there are no comprehensive papers on TA, only case reports. No reports on TTM for TA patients are available. We herein describe a case of TA that resulted in decorticate posturing, but a good neurological outcome was achieved with TTM.

## Case presentation

The patient was a 59-year-old male and a transportation worker. He was caught between a 4-ton steel plate and a container for about five minutes during a transport operation. A helicopter emergency medical service was called. When the flight physician examined the patient, his airway was open and his breathing and circulation were stable, but his Glasgow Coma Scale (GCS) was E1V1M3 with decorticate posturing. The patient was intubated on-site and transported by air to our hospital.

**Figure 1:**
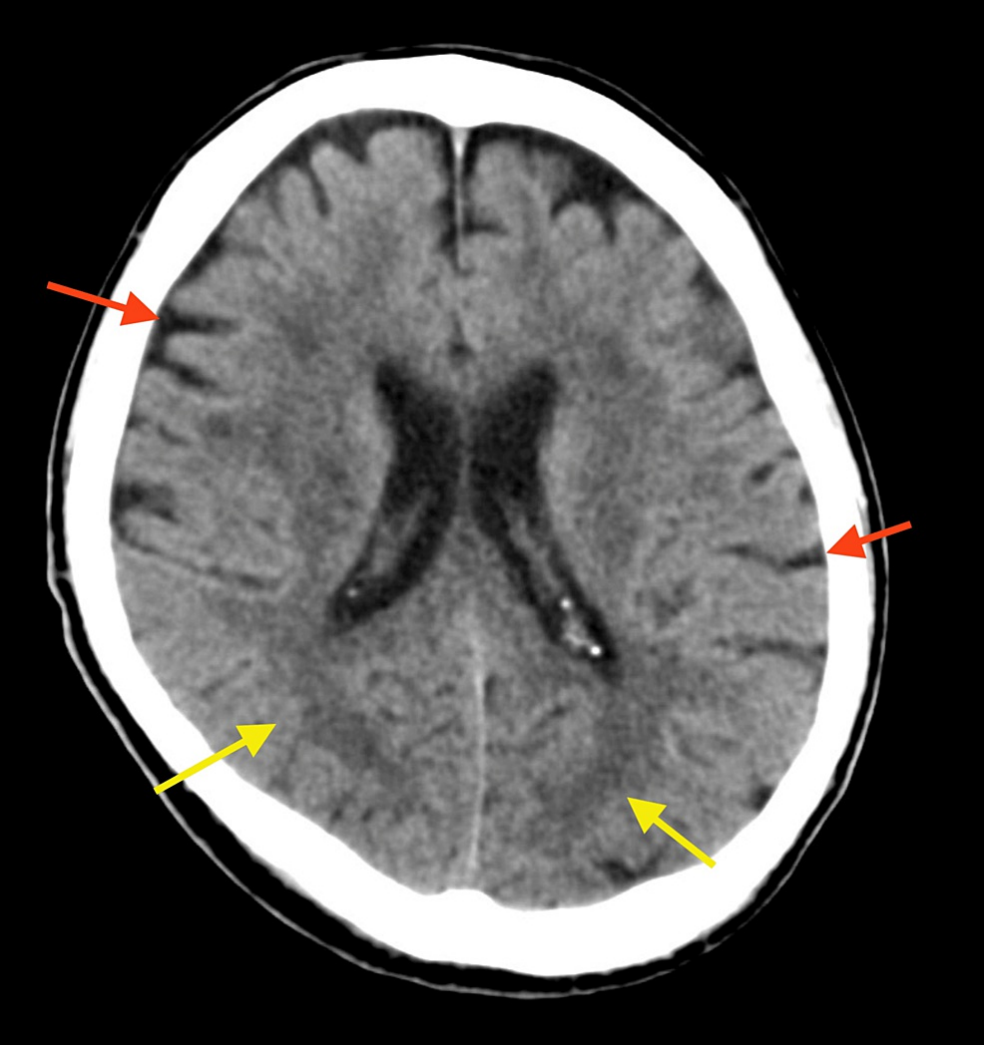
Head CT scan on arrival. The corticomedullary boundary is clear as shown by the yellow arrows, and the cerebral sulcus is not narrowed as shown by the red arrows. CT: computed tomography

He was hemodynamically stable and no more resuscitation was required. Computed tomography (CT) scan revealed no TBI. Corticomedullary borders were visible, and there was no narrowing of the cerebral sulcus (Figure 1).

Although left traumatic pneumothorax and liver injury were noted, both of which did not require surgical intervention. Since the patient was considered to be unconscious due to hypoxic encephalopathy associated with TA, TTM was performed with Gaymar Medi-Therm III™. Because he had multiple traumas such as liver injury, normothermia was chosen.

TTM was completed in 24 hours at 36°C. He was weaned from the ventilator and extubated on the third day. After extubation, the patient was able to communicate simply and express his own intentions in a simple way such as nodding or shaking hands. Thereafter, his behavior became increasingly inconsistent, and a psychiatrist diagnosed him with hypoxic encephalopathy or delirium. The delirium subsided over time, and after continued rehabilitation for higher brain functions, the Mini-Mental State Examination (MMSE) score improved markedly from 12 points on the seventh day to 27 points on the 12th day.

**Figure 2:**
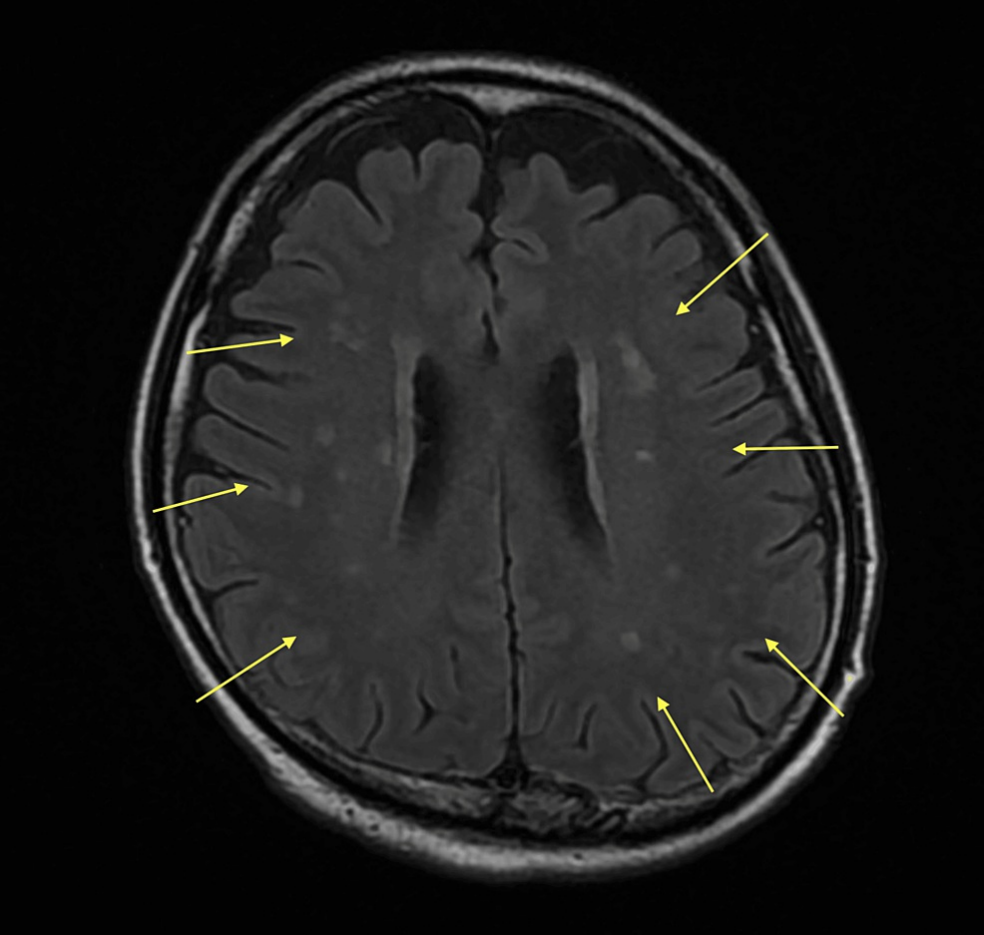
T2-weighted MR image of the head on the 15th day after injury. The yellow arrows show scattered high-signal areas in the brainstem, bilateral basal ganglia regions, and periventricular white matter of the lateral ventricles. MR: magnetic resonance

Magnetic resonance imaging (MRI) taken on the 15th day showed scattered high-signal areas in the brainstem, bilateral basal ganglia regions, and periventricular white matter of the lateral ventricles, which appeared to be infarct ischemic foci (Figure 2). Whole-brain ischemia might occur during the pincer pressure trauma, and the perforating branch regions were infarcted. The patient was transferred to a rehabilitation hospital on the 22nd day to continue rehabilitation for higher brain dysfunction. At the time of transfer, his GCS had been recovered to 15.

## Discussion

TTM is primarily used after cardiopulmonary resuscitation for the purpose of cerebral protection [5].

TTM is not commonly performed in trauma patients because hypothermia can exacerbate traumatic coagulopathy and lead to a poor prognosis. On the other hand, fever may be associated with poor neurological prognosis, and TTM might improve it. There are scattered reports of its usefulness in patients with severe TBI [7]. To the best of our knowledge, this is the first reported case of TA which underwent TTM with a good neurological outcome.

TA is often associated with severe trauma to the trunk, and hypothermia is contraindicated in such cases because it causes coagulopathy and interferes with hemostasis. Thus, normothermia is often the treatment of choice in TTM for trauma patients [5]. In our present case, the patient also suffered multiple rib fractures, traumatic pneumothorax, and liver injury, none of which required surgery. We too opted for TTM at 36°C for 24 hours.

Although decorticate rigidity was observed at the time of transport (GCS E1V1M3), the patient was found to follow our instructions after TTM, and finally, his GCS became 15.

Although loss of consciousness was observed immediately after the injury, the patient recovered without major cerebral disturbance, only with higher brain dysfunction such as memory impairment and attention disorder. The effect of TTM was sufficient in our case.

## Conclusions

TA is a very rare condition, and there is no standardized management protocol. TTM is not commonly used in trauma patients because of concerns about coagulopathy, and its utility has only been suggested for TBI. In our case, we performed TTM on a TA patient and obtained a good outcome. TTM can be a useful option in TA patients with no other severe hemorrhagic trauma.
